# Glyphosate toxicity and carcinogenicity: a review of the scientific basis of the European Union assessment and its differences with IARC

**DOI:** 10.1007/s00204-017-1962-5

**Published:** 2017-04-03

**Authors:** Jose V. Tarazona, Daniele Court-Marques, Manuela Tiramani, Hermine Reich, Rudolf Pfeil, Frederique Istace, Federica Crivellente

**Affiliations:** 1 Pesticides Unit, European Food Safety Authority, Via Carlo Magno 1/A, 43126 Parma, Italy; 2 Federal Institute for Risk Assessment (BfR), Berlin, Germany

**Keywords:** Glyphosate, Toxicity, Carcinogenicity, IARC, EFSA, Public health, Consumer risk

## Abstract

Glyphosate is the most widely used herbicide worldwide. It is a broad spectrum herbicide and its agricultural uses increased considerably after the development of glyphosate-resistant genetically modified (GM) varieties. Since glyphosate was introduced in 1974, all regulatory assessments have established that glyphosate has low hazard potential to mammals, however, the International Agency for Research on Cancer (IARC) concluded in March 2015 that it is probably carcinogenic. The IARC conclusion was not confirmed by the EU assessment or the recent joint WHO/FAO evaluation, both using additional evidence. Glyphosate is not the first topic of disagreement between IARC and regulatory evaluations, but has received greater attention. This review presents the scientific basis of the glyphosate health assessment conducted within the European Union (EU) renewal process, and explains the differences in the carcinogenicity assessment with IARC. Use of different data sets, particularly on long-term toxicity/carcinogenicity in rodents, could partially explain the divergent views; but methodological differences in the evaluation of the available evidence have been identified. The EU assessment did not identify a carcinogenicity hazard, revised the toxicological profile proposing new toxicological reference values, and conducted a risk assessment for some representatives uses. Two complementary exposure assessments, human-biomonitoring and food-residues-monitoring, suggests that actual exposure levels are below these reference values and do not represent a public concern.

**Electronic supplementary material:**

The online version of this article (doi:10.1007/s00204-017-1962-5) contains supplementary material, which is available to authorized users.

## Introduction

Glyphosate is the most widely used herbicide in the world. A broad spectrum herbicide, its uses include weed control in agriculture, vegetation control in non-agricultural areas, and harvesting aid as crop desiccant. Its use in agriculture has increased considerably due to the development of glyphosate-resistant GM crop varieties; the herbicide has also been used to control illegal crops through massive aerial applications (Solomon et al. 2007). The widespread use and public debate regarding these uses have aroused societal concern and a scientific controversy on the toxicity of glyphosate (Faria 2015) beyond the scientific debate (Blaylock 2015).

Glyphosate was considered an advantageous herbicide until its use led to the evolution of glyphosate-resistant weeds (Duke and Powles 2008) and studies suggesting effects of glyphosate-based formulations in humans and wildlife were published. Interest in glyphosate has increased exponentially among scientists, and the subject accounted for 5% of the articles on pesticides included in PubMed during 2015. About 25% of the articles cover the toxicity endpoints in humans and all types of organisms, and the majority is conducted with glyphosate-based formulations, containing other ingredients. Some ingredients may be more toxic than glyphosate for non-plant species (Kim et al. 2013; Mesnage et al. 2013; Nobels et al. 2011), ingredients classified as carcinogenic or mutagenic are not expected to be used and must be indicated in the label, however, the full composition of the formulation is not disclosed by the manufacturers, therefore, it is impossible for researchers to apply mixture toxicity methods and attribute toxicity to specific ingredients.

The risk assessment of a pesticide for human health integrates two aspects. First, the hazard identification clarifies the toxicological profile of the substance, setting the type of health effects it is expected to produce in humans depending on the level of exposure, triggering the hazard classification and setting the toxicological reference values to be used in the risk assessment. Then, for each intended use, the expected level of exposure is calculated and compared with the reference values. While the hazard potential is intrinsic and, therefore, expected to be equivalent in all evaluations, the risk is related to the use of the substance—which is defined as the likelihood and magnitude of adverse effects—and strongly depends on the patterns and conditions of use.

Glyphosate has been the subject of regular assessments by national and international regulatory agencies (JMPR 2006; Williams et al. 2000). All had established that glyphosate has a relatively low toxicity in mammals. However, a recent report from the International Agency for Research on Cancer (IARC) concluded that the herbicide and its formulated products are probably carcinogenic in humans (Guyton et al. 2015a, b; IARC 2015). The aim of IARC’s assessments is to identify carcinogenicity hazards as a first step in carcinogenic risk assessment. IARC assessments do not include recommendations regarding regulatory or legislative decisions; they are scientific evaluations informing regulatory assessments. Consequently, the IARC conclusion triggered a reconsideration of the evidence on carcinogenicity in the EU evaluation, and more recently by the Joint FAO/WHO Meeting on Pesticide Residues. The European Union renewal process (European Food Safety Authority 2015a, b; Germany 2015) was the first comprehensive regulatory assessment of glyphosate conducted after the IARC evaluation. Following a detailed assessment of all available information, the European assessment reached a different conclusion, increasing the scientific and social debate. In 2016 the Joint FAO/WHO Meeting on Pesticide Residues concluded that glyphosate is not carcinogenic in rats but could not exclude the possibility that it is carcinogenic in mice at very high doses, this information was used in the risk assessment concluding that glyphosate is unlikely to pose a carcinogenic risk to humans from exposure through the diet (JMPR 2016). This manuscript explores possible reasons for the different conclusions, with a focus on the EU assessment, as this is the evaluation in which the authors have been involved.

Typically, regulatory assessments come to conclusions similar to those of IARC, but there are exceptions (Pearce et al. 2015). Scientific divergences may result from different sets of evidence, different approaches and methods, or different interpretations when weighing ambiguous results. Divergences are particularly likely when one evaluation includes additional evidence. In this context, it is important to mention that the EU evaluation, which considered studies not available to IARC, also updated the toxicological profile of glyphosate, proposing new toxicological reference values.

IARC monographs cover carcinogenicity hazard identification. When statistical associations between exposure and cancer incidences are observed in epidemiological studies, the assessment of causal relationships may lead to divergent conclusions (Rhomberg 2015a, b). The comparison of both glyphosate assessments is used below to explain the different aims, methods and possible divergences between regulatory and IARC assessments—focusing on the glyphosate carcinogenicity hazard identification as a case study—and, more importantly, their role in the assessment of risks to consumers and public health concerns. The example is particularly useful as both evaluations were conducted within the same period, and as the EU assessment, based on the United Nations Globally Harmonised System (UN-GHS) for classification of chemicals, is also relevant in the broad international context.

## Methodology: scientific assessment of carcinogenicity and its use in the regulatory context

Pesticides are heavily regulated chemicals and require pre-marketing authorisation in most jurisdictions. The EU system also includes a renewal process, requiring all pesticides to be regularly re-assessed in the light of new scientific developments and information requirements. The EFSA assessment (European Food Safety Authority 2015b) followed an evaluation carried out by the European Commission in 2002.

The identification of carcinogenic chemicals and carcinogens in food is of high societal and scientific interest (Barlow and Schlatter 2010). The communication of the outcome of the risk assessment is complex and controversial in the case of equivocal results (Downes and Foster 2015). The identification of a mutagenic or genotoxic mechanism plays an important role in risk assessment and requires a critical evaluation of the data as well as expert judgment (Eastmond 2012). The hazard assessment is linked to the classification; the EU uses the hazard assessment system for chemicals developed by the United Nations following the 1992 UN Earth Summit (Pratt 2002). This Globally Harmonised System for classifying chemicals replaces previous national and international approaches, is specifically recommended by FAO to be used for pesticides, and is implemented in the EU Classification, labelling and packaging (CLP) regulation—(EC) No 1272/2008—and other jurisdictions (UNECE http://www.unece.org/trans/danger/publi/ghs/implementation_e.html).

IARC and regulatory assessments are usually complementary. The different roles, methods and information sources of IARC and regulatory assessments, as well as the implications for public health, must be considered in case of divergences and are summarised in Table 1. IARC identifies carcinogenic hazards resulting from occupational, environmental, and lifestyle exposures and agents as a first step of the risk assessment process, and has developed an internationally recognised grouping system that includes defined criteria and methodology (Guyton et al. 2015a, b; Lauby-Secretan et al. 2016; Pearce et al. 2015; Straif et al. 2014). The recently developed approach for assessing mechanistic information, based on the characteristics of IARC group 1 carcinogens, was applied for glyphosate (Smith et al. 2016). Regarding data sources, IARC assessments are primarily based on published evidence, i.e. scientific publications and regulatory assessments; industry-sponsored studies are used when reviewed and reported in regulatory evaluations, becoming a relevant secondary source for regulated agents such as pesticides. Both, scientific publications and mandatory industry-sponsored studies, were primary sources in the EU evaluation.

**Table 1:** Comparison of IARC and regulatory assessments roles and methodological elements

Issue	IARC	EU regulatory assessment
Role	Hazard based identification. First step to be used by authorities in their risk assessments. No regulatory power	Scientific assessment covering hazard identification (classification), hazard characterisation (setting toxicological reference values), exposure assessment, and risk characterisation Formal support for decision making
Coverage	IARC selection, based on criteria such as identified concern or human exposure. Chemical, physical, biological or behavioural “agents” 58 pesticides	Mandatory, 1355 pesticide active substances in the EU data base. Chemical and microbial pesticides
Method	IARC developed methodology, described in the “preamble”. Applicable to all agents	For chemical pesticides, hazard identification based on UN GHS criteria Detailed guidance from ECHA available
Sources	Review of published information. Summaries of industry sponsored studies used as secondary source if obtained from regulatory agency reports	Full set of mandatory (OECD guidelines) GLP studies and epidemiological data Review of scientific peer-review publications, last 10 years Information collected through a public consultation
Formulations	“Agent” grouped as active substance and all formulated products together	UN GHS principles applied to the active and then to each formulation, accounting for all other ingredients

For pesticides, IARC identifies the “carcinogenic agent” as the active pesticide substance and its commercial formulations; the specific role of the other formulation ingredients in the occurrence of effects is not considered separately from the active ingredients. This is in line with the role of human evidence in IARC assessments. Epidemiological studies of farmers and consumers have very limited information on actual exposure levels (Ntzani et al. 2013), and use the pesticide active substance as descriptor, combining individuals exposed to different formulations without discriminating the different compositions. In the regulatory context, each formulation should be assessed according to its composition, identifying the role of the active substance and of the other ingredients; and the risk management measures are set for the chemical responsible for the effect, either active substance or co-formulant.

The UN-GHS and IARC frameworks use different terminology, but the definitions for sufficient and limited evidence in humans and in animals are similar and can be used to establish equivalences between both schemes, as presented in Table 2.

**Table 2:** Proposed equivalences between the UN-GHS and IARC classification schemes

	*Category 1A*	*Category 1B*	*Category 2*	*No classification*	
UN-GHS and CLP	Substances known to have carcinogenic potential for humans Largely based on human evidence	Substances presumed to have carcinogenic potential for humans Largely based on animal evidence	Substances suspected to have carcinogenic potential for humans Evidence obtained from human and/or animal studies but not sufficiently convincing to place the Substance in Category 1A or 1B	No sufficient evidence for classifying the substance as carcinogenic	
	*Group 1*	*Group 2A*	*Group 2B*	*Group 3*	*Group 4*
IARC	The agent is a carcinogen for humans. This category is only used when sufficient indications of carcinogenicity for humans are available	The agent is probably carcinogenic for humans. The classification of an agent in this category is recommended if there is no formal evidence of carcinogenicity in humans, but corroborating indicators of its carcinogenicity for humans and sufficient evidence of carcinogenicity in experimental animals	The agent is possibly carcinogenic for humans. There is limited evidence of carcinogenicity in humans and evidence for animals, or insufficient evidence for human beings but sufficient evidence of carcinogenicity in experimental animals	Agent not classifiable as to its carcinogenicity to humans. (Insufficient evidence for human beings and insufficient or limited for animals)	Agent probably not carcinogenic for humans. (Evidence suggesting lack of carcinogenicity in humans and in experimental animals)

This approach allows a comparison of the pesticides evaluated by IARC with the current EU classification (Table 3 and supplementary material Annex 1). The EU classification includes scientific assessments conducted by the European Chemicals Bureau of the European Commission—some, but not all, based on EFSA evaluations—and by the Committee for Risk Assessment of the European Chemicals Agency.

**Table 3:** Overall comparison of the carcinogenicity assessments of pesticides conducted by EFSA and IARC (see supplementary material for information on the pesticides classified in each category)

	*Category 1A*	*Category 1B*	*Category 2*	*No classification*		*Not assessed/no data*
EU	0	17	53	30		4
	*Group 1*	*Group 2A*	*Group 2B*	*Group 3*	*Group 4*	*Not assessed*
IARC	3	8	13	34	0	56

A total of 53 pesticides have been assessed under both systems. For about half—29 out of 53—the classifications are equivalent; the EU classification is more severe/conservative for 14 pesticides and less severe/conservative for 11. It should be noted that 8 out of the 11 pesticides with more severe/conservative classification by IARC are those assessed in recent IARC monographs. New substances are evaluated and others re-evaluated regularly, leading to changes in the classification; thus the table represents just a “screen-shot” of two rolling processes. Differences with IARC and between jurisdictions have also been reported for other regulatory assessments (Choi and Lim 2010). Both IARC and regulatory classifications are based on the information available at the time of the evaluation. For pesticides, the identification of possible concerns triggers the generation of additional evidence and a subsequent evaluation; consequently, some differences are not real scientific divergences but the result of expert re-evaluations based on different sources of evidence. This may have played a role in the case of glyphosate, as discussed below.

## Discussion

### Understanding the divergence: glyphosate carcinogenicity assessment

The carcinogenicity of glyphosate has been reviewed by several national and international agencies (Ibrahim 2015). The outcome of the EU assessment, the differences with the IARC evaluation (IARC 2015), and the authors’ views explaining these differences, are summarised below. Additional details are provided in the supporting information.

#### Human evidence

IARC (2015) offered the most up-to-date review of human epidemiological studies on glyphosate. Positive evidence regarding an association between exposure to glyphosate and non-Hodgkin lymphoma, observed in some case-control studies but not confirmed by cohort studies, was considered sufficient by IARC to conclude on “limited evidence” in humans. Limited evidence is defined as a positive association observed between exposure to the agent and cancer, for which a causal interpretation is considered to be credible, but chance, bias or confounding could not be ruled out with reasonable confidence. This definition was developed by IARC and introduced in the UN-GHS criteria (United Nations 2003) and EU Regulation (EC) No 1272/2008. EFSA re-assessed the same information; the association with non-Hodgkin lymphoma was discussed during an expert meeting. The statistically significant association was considered limited due to low power, lack of consistency, and the view that greater weight should be given to the cohort study for non-rare tumours. Considering causality, the majority of the experts concluded that the epidemiological evidence was very limited, and insufficient for classification. Although the role of the weight attributed to case–control studies versus cohort studies cannot be fully ruled out, the main reason for the divergent views could be the possibility of bias, chance results and confounding effects, as IARC concluded that the limited evidence in humans was supported by sufficient evidence of carcinogenic potential in animals and strong mechanistic evidence for genotoxicity and oxidative stress. As explained below, the EU evaluation used additional evidence regarding animal carcinogenicity and genotoxicity, and reached different conclusions.

#### Carcinogenicity in animals

##### Information sources

There is only one published study on the carcinogenicity of the active substance glyphosate in rats (Chruscielska et al. 2000), which showed no significant increase in tumour incidences in any treated group. Two additional published studies on glyphosate formulations, the first one on initiation-promotion in mice (George et al. 2010) and the second one, a study of rats (Seralini et al. 2014) that was retracted and republished creating some controversies (Fagan et al. 2015), were considered inadequate by IARC and EFSA for carcinogenicity assessment (European Food Safety Authority 2012; IARC 2015). Consequently, industry-sponsored studies, required by several jurisdictions worldwide, have constituted the basis for the assessment of animal carcinogenicity by both IARC and EFSA. As expected for a regulatory assessment, EFSA assessed the original study reports. According to their principles, IARC used unpublished studies based on secondary sources, i.e. the information on the studies as published by JMPR (2004) and US-EPA (1993). The time difference, over a decade, between the IARC monograph and the published regulatory assessments must be considered. Five new studies, not assessed by the JMPR and US-EPA, and therefore, not considered by IARC, were considered valid and included in the EU assessment. The IARC assessment is based on the re-assessment of industry-sponsored studies, two in mice and four studies in rats, plus the negative published study in rats. The EU assessment included five additional valid studies, two in mice and three in rats; one mouse study was excluded due to a likely viral infection in the experimental population and one rat study was considered inadequate due to study deficiencies. Table 4 summarises the studies used in the EU assessment; additional information is provided in Table S-2 as supplementary material, with links to the detailed summaries for each study and its assessment as published in the EFSA background document (Germany 2015). Additional information and raw data have been published as supplementary information in a recent industry-sponsored review of glyphosate carcinogenicity (Greim et al. 2015).

**Table 4:** Review of long-term chronic toxicity and carcinogenicity studies considered during the EU assessment

Study reference—Authors	Duration, strain, study type	Dose levels (NOAEL/LOAEL) mg/kg bw per day	Critical effect at the LOAEL
Mice long-term chronic toxicity and carcinogenicity studies used in the EU evaluation			
A - Knezevich and Hogan (1983)	2 year, CD-1, OECD TG 451/453	0, 157, 814, 4841 (157/814)	Males: body weight reduction, hepatocellular centrilobular hypertrophy and bladder epithelial hyperplasia
B - Atkinson et al. (1993)	2 year, CD-1, OECD TG 451	0, 100, 300, 1000 (1000/>1000)	Equivocal enlarged/firm thymus, not associated with histopathological findings (considered not biologically relevant)
C - Sugimoto (1997)	18 month, CD-1 (ICR), OECD TG 451	0, 153, 787, 4116 (153/787)	Body weight gain, reduction food consumption and efficiency, loose stool, caecum distended and increased weight, prolapse and anus ulceration
D - Wood et al. (2009)	18 month, CD-1 (ICR), OECD TG 451	0, 71, 234, 810 (810/>810)	No adverse effects observed
Rat long-term chronic toxicity and carcinogenicity studies used in the EU evaluation			
E - Lankas (1981)	26 month, Sprague–Dawley rat, combined chronic toxicity/carcinogenicity;, Not Good Laboratory Practice (GLP) compliant	0, 3, 10.3, 31.5 (31.5/>31.5)	No adverse effects observed*
F - Stout and Ruecker (1990)	2 year, Sprague–Dawley rat, US-EPA F 83 − 5	0, 89, 362, 940 (89/362)	Reduction body weight and gain, increase liver weight, stomach mucosal inflammation, cataracts, decrease urine pH, survival <50% in all groups incl. controls
G - Atkinson et al. (1993)	2 year, Sprague–Dawley rat, US-EPA F 83 − 5	0, 10, 100, 300, 1000 (100/300)	Pronounced salivary gland findings, increase AP and liver weight
H - Suresh (1996)	2 year, Wistar rat, OECD TG 453	0, 6.3, 59.4, 595.2 (60/595.2)	Cataracts, increase AP
I - Lankas 1997	12 month, Wistar rat, OECD TG 452	0, 141, 560, 1409 (141/560)	Reduction in body weight, food cons and utilization, increase AP, focal basophilia of acinar cells of parotid salivary gland (not weighed)
J - Enomoto (1997)	2 year, Sprague–Dawley rat, OECD TG 453	0, 104, 354, 1127 (104/354)	Reduction body weight, gain, food cons (initially) and utilization, increase loose stool, increase tail masses due to follicular hyperkeratosis and abscesses, caecum: distension and increase weight, pH reduction and dark appearance of urine
K - Brammer (2001)	2 year, Wistar rat, OECD TG 453	0, 121, 361, 1214 (361/1214)	Reduction body weight, food cons and (initially) utilization, clinical chemistry findings (increase AP and ALAT activity and bilirubin, decrease urine pH), kidney papillary necrosis, prostatic and periodontal inflammation
L - Wood et al. (2009)	2 year, Wistar rat, OECD TD 453	0, 86, 285, 1077 (285/1077)	Reduction body weight gain, transient increase AP, changes in distribution of renal mineralisation, increase adipose infiltration of bone marrow (indicative of hypoplasia)
M - Chruzielska et al., 2000	24 month, Wistar rat, in drinking water	0, 300, 900 or 2700 mg/L	No significant increase in tumour incidence
Industry-sponsored GLP studies considered non-acceptable during the EU assessment			
N - Kumar (2001)**	18 month, Swiss albino mice, OECD TG 451	Title: Carcinogenicity Study with Glyphosate Technical in Swiss Albino Mice	
O - Bhide (1997)***	2 year Sprague–Dawley rat, OECD TG 453	Title: Combined Chronic Toxicity/Carcinogenicity Study of Glyphosate Technical in Sprague Dawley Rat	
Published studies conducted with glyphosate-based formulations and considered non-reliable for the assessment of glyphosate carcinogenicity during the EU assessment			
P - George et al. (2010 carcinogenicity)	Non-guideline mechanistic study conducted with topical application of glyphosate-based formulation	Title: Studies on glyphosate-induced carcinogenicity in mouse skin: A proteomic approach	
Q - Seralini et al. (2012), re-published 2014	24-month study (10 males and 10 females per group) Sprague–Dawley rats in drinking water	Title: Long term toxicity of a Roundup herbicide and a Roundup-tolerant genetically modified maize	

*The dose levels used in this study are too low and the study is not considered adequate to assess glyphosate chronic toxicity/carcinogenicity

**Study N found unreliable after detailed assessment, due to the occurrence of viral infection in all groups including controls

***Study O was considered not acceptable because no core information on the test substance such as batch number or purity was given and, thus, it is not clear what was in fact tested. Furthermore, the study presented many deficiencies

##### Assessment of the available evidence

In its weight of evidence, the IARC Working Group considered a statistically significant trend for renal tumours in male mice in one study (study A in Tables 4, 5) and for haemangiosarcoma in the other (study B in Tables 4, 5). No statistically significant increase in tumour incidence in females was observed in these studies. In the weight of evidence in rats, the IARC Working Group considered increases in the incidence of adenomas, with no evidence of progression to carcinomas, in pancreatic islet cells in males (studies E and F in Table 4), hepatic cells in males (study E in Table 4) and thyroid C-cell in females (study E in Table 4). No increase in tumour incidence was observed in three studies (studies G, K and M in Table 4). The EU assessment followed the weight of evidence approach required by the UN-GHS criteria (United Nations 2015) and further clarified in the ECHA guidance (European Chemicals Agency 2015). The statistical significance found in trend analysis in some studies was balanced against the lack of statistical significance in pair-wise comparison tests, lack of consistency in multiple animal studies, slightly increased incidences only at dose levels at or above the Maximum Tolerable Dose (MTD), lack of pre-neoplastic lesions and/or whether the studies fell within the relevant historical control range. A specific comparison of tumour incidences in male CD-1 mice from four carcinogenicity studies (no change in tumour incidence was observed in females) is provided in Table 5, and the detailed scientific assessment and weight of evidence for each tumour type is summarised in Table 6.

**Table 5:** Summary of selected tumour incidences in male CD-1 mice from four studies with glyphosate and historical control data

Dose range	Tumour incidence/number of animals examined															
	Control group				Low dose				Intermediate dose		High dose				Very high dose***	
Dose (mg/kg bw per day)	0	0	0	0	71	100	157	165	234	300	810	814	838	1000	4348	4841
Study ID	A	B	C	D	D	B	A	C	D	B	D	A	C	B	C	A
Study duration (months)	24	24	18	18	18	24	24	18	18	24	18	24	18	24	18	24
Survival	20/50	26/50	26/50	39/51	41/51	25/50	16/50	34/50	39/51	29/50	35/51	17/50	27/50	25/50	29/50	26/50
Renal tumours^#^	1/49	2/50	0/50	0/51	0/51	2/50	0/49	0/50	0/51	0/50	0/51	1/50	0/50	0/50	2/50	3/50
Malignant lymphoma*	2/48	4/50	**2**/**50**	0/51	1/51	2/50	5/49	**2**/**50**	2/51	1/50	5/51	4/50	**0**/**50**	6/50	**6**/**50**	2/49
Haemangiosarcoma**	0/48	**0**/**50**	0/50	2/51	1/51	**0**/**50**	0/49	0/50	2/51	**0**/**50**	1/51	1/50	0/50	**4**/**50**	2/50	0/49

Study ID: A = TOX9552381 (1983), PWG re-evaluation; B = TOX9552382 (1993); C = ASB2012-11493 (1997); D = ASB2012-11492 (2009)

*Study A: Malign lymphoblastic tumours (3 categories) instead of malignant lymphoma which was not mentioned as a pathological entity

**Whole body/multiple organ

***Dosage exceeded the OECD-recommended limit dose of 1000 mg/kg bw/day and the MTD

Numbers in bold refer to values within acceptable HCD; no HCD is available for the other values (not bold) and no exceedance of HCD was recorded in mice treated with glyphosate

^#^Renal tumours: combined incidence of adenoma and carcinoma

**Table 6:** Summary of the weight of evidence of the EU assessment for the different tumour types

Tumour type/species	Significant trends	Weight of evidence in EU assessment
Renal tumours, mice	2 out of 4 studies TOX9552381 (6% combined adenomas and carcinomas in males at 4841 mg/kg bw day) ASB2012-11493 (4% adenomas in males at 4348 mg/kg bw day)	Both studies, trends observed only at high dose (>4000 mg/kg bw per day), where general toxicity (such as reduced bw, histopathological findings in liver, and bladder in one study and reduced bw gain, severe gastro-intestinal effects in the other) may be confounding factors No statistical significance in a pair-wise comparison One trend in one study did not consider the higher survival at the top dose
Malignant lymphomas, mice	2 out of 4 studies ASB2012-11493 (12% males at 4348 mg/kg bw day) ASB2012-11492 (10% males at 810 mg/kg bw day)	Malignant lymphomas is one of the most common neoplasms in CD-1 mice, females being more prone to this tumour type than males No statistical significance in a pair-wise comparison First study within historical controls and trend observed only at high dose (>4000 mg/kg bw per day), where general toxicity may be a confounding factor Second study inconsistency in results among 4 studies comparing similar dose levels
Haemangiosarcomas, mice	2 out of 4 studies TOX9552382 (8% males at 1000 mg/kg bw day) ASB2012-11493 (4% males at 4348 mg/kg bw day)	No statistical significance in a pair-wise comparison First study within historical control range Second study trend observed only at high dose (>4000 mg/kg bw per day) where general toxicity may be a confounding factor
Hepatocellular adenomas, rats	1 out of 8 studies TOX9300244 (15% males at 940 mg/kg bw day)	No statistical significance in a pair-wise comparison Marginal trends in benign tumours limited to one sex, not reproduced among 8 long term studies (3 studies in SD rats and 5 studies in Wistar rats)
Thyroid C-cell adenomas, rats	1 out of 8 studies TOX9300244 (10% females at 457 and 1183 mg/kg bw day)	No statistical significance in a pair-wise comparison Marginal trends in benign tumours limited to one sex, not reproduced among 8 long term studies (3 studies in SD rats and 5 studies in Wistar rats)
Pancreatic islet cell adenomas, rats	Incidences without dose response trends in 2 out of 8 studies	Lack of dose–response does not support an effect related to glyphosate administration
All other tumours, mice and rats	No increased incidences observed in 4 mice and 8 rat studies	No observed incidences in a large number of valid studies

##### Comparison of both weight of evidence approaches

As indicated by Portier et al. (Portier et al. 2014), individual scientific studies are rarely, if ever, conclusive. In our view, this is particularly relevant when assessing the carcinogenicity potential in humans using animal studies, and supports the need for a consistency check combining all available studies as mandated in the UN-GHS criteria.

In the absence of conclusive human evidence, and despite some views suggesting the need for re-assessing its relevance (Beyer et al. 2011; Marone et al. 2014; Osimitz et al. 2013), rodent long-term toxicity/carcinogenicity studies are used for predicting carcinogenicity in humans (Doktorova et al. 2012). False positives and false negatives should both be considered, weighing the evidence (Lutter et al. 2015; Rhomberg 2015a, b; Rhomberg et al. 2013) and assessing specifically human relevance; and linked to the MTD concept, the relevance of toxicity-induced carcinogenic effects observed in experimental animals only at very high doses. The UN-GHS, and therefore, the EU CLP approach are based on UN harmonised criteria for weighing the evidence from rodent studies. Regulatory (European Chemicals Agency 2015) and non-regulatory (McGregor et al. 2010) guidance is available for weighing the evidence in line with the UN-GHS criteria. Table 7 summarises the assessment of the different UN-GHS Weight of Evidence elements in the EU assessment, and includes a comparison with the weight provided in the IARC evaluation. It should be noted that the authors of this paper did not participate in the IARC assessment, and therefore, the IARC columns are based on the information extracted from the IARC preamble and monograph, and do not reflect the Working Group discussions except when specifically reported in the monograph. The elements detailed in Tables 5, 6 and 7, and used in the EU evaluation, are not only specific components of the regulatory guidance (European Chemicals Agency 2015), but, as described below, are also fully supported by current scientific knowledge on the assessment of animal studies.

**Table 7:** Summary of the UN-GHS Weight of Evidence (WoE) elements in the EU assessment and comparison with the weight provided in the IARC assessment

UN-GHS and EU CLP WoE elements	Regulatory guidance (ECHA, 2015) and scientific support	Evaluation method in the IARC Preamble	Relevance for the glyphosate WoE	
			EU assessment	Comments on IARC assessment
(a) Tumour type and background incidence	Relevance for humans, due to the relevance of the mode of action(Meek et al. 2014a, b), or tissues with no human equivalents. Spontaneous incidences and use of historical control data(Dinse and Peddada 2011; Greim et al. 2003; Keenan et al. 2008; Ma et al. 2002; Massarelli et al. 2013)	Relevance for humans, e.g. species-specific mechanism that does not operate in humans The use of historical data is mentioned	All valid studies are considered negative. No need for mode of action evaluation Historical control data from the same laboratory were considered when available	All tumours were assumed relevant for humans No information on the use of historical control data is provided except the consideration of some tumours as “rare”
(b) Multi-site responses	If observed, increases the evidence (Dybing et al. 1997)	Consistency of the results across target organ(s) and spectrum of neoplastic response	No significant incidences observed in the valid studies Consistency among studies was considered	Based on statistically significant trends for different tumour types. Assessment limited to a subset of the available studies
(c) Progression of lesions to malignancy	If observed, increases the evidence	The spectrum of neoplastic response, from preneoplastic lesions and benign tumours to malignant neoplasms	Specifically considered for individual studies	Specifically considered for individual studies
(d) Reduced tumour latency	Only relevant for unusual tumours	Sufficient for considering the agent as carcinogen	Not relevant	No indications are provided
(e) Whether responses are in single or both sexes	A consistent mode of action is required for tumours observed in only one sex	No specific indications for the evaluation of tumours occurring in a single sex are provided	Contributes to the lack of consistency assessment as no sex related mode of action is postulated	All trends were significant only in one sex, but no sex mediated mode of action is discussed
(f) Whether responses are in a single species or several species	If observed in several species increases the evidence	If observed in several species increases the evidence	No significant incidents were identified for mice or rats	Based on positive trends in both mice and rats
(g) Structural similarity to a substance(s) for which there is good evidence of carcinogenicity	Includes SAR, QSAR, read across and grouping	The possibility for using information from structurally similar agents is mentioned	Not relevant, the assessment is based on studies on glyphosate	Not relevant, the assessment is based on studies on glyphosate
(h) Routes of exposure	Includes local tumours	The exposure route should be mentioned	Assessment based on oral studies	Assessment based on oral studies
(i) Comparison of absorption, distribution, metabolism and excretion between test animals and humans	Also relevant for considering the role of metabolites	Comparison should be made when possible	Not relevant	Not relevant
(j) The possibility of a confounding effect of excessive toxicity at test doses	Effects observed only at doses exceeding the maximum tolerable dose should be checked for confounding effects of excessive toxicity	Not mentioned in the preamble. NOAELs and LOAECs for each study are not reported	Considered for tumours in mice	Effects observed only at high doses with excessive toxicity are included in the trend assessment. No additional information is provided
(k) Mode of action and its relevance for humans, such as cytotoxicity with growth stimulation, mitogenesis, immunosuppression, mutagenicity	The IPCS framework and related approaches (Boobis et al. 2006, 2008; Meek et al. 2014a; Sonich-Mullin et al. 2001) offers general guidance, IARC (1994, 1999) and ECHA (2015) list specific tumours considered not relevant for humans. Mutagenicity and genotoxicity play a key role in the assessment and in particular the assessment of non-threshold genotoxic-carcinogenic modes of action	The possible mechanism should be identified when possible. The assessment of genotoxicity is described in the preamble, *in vivo* data on humans and mammals have preference. No mention to the “ten key characteristic approach” is included in the preamble	The genotoxicity assessment is based on mammalian studies, and concluded as negative for glyphosate, as all studies are negative except at very high doses with confounding cytotoxicity. Genotoxicity of a co-formulant and of some glyphosate formulations cannot be ruled out, and should be addressed	The conclusion of strong evidence on genotoxicity and oxidative stress for glyphosate and glyphosate based formulations is one of the key arguments of the IARC proposal. The differences between glyphosate and glyphosate based formulations reported in several studies are presented but no further discussed

Due to the large number of studies, the assessment of chance results is particularly relevant. Dose–response within the study, consistency among similar studies, consistency or justified differences between sexes, and comparison with historical controls, are considered key elements for identifying chance effects. The Bradford Hill guidelines published in 1965 are still considered a reference for assessing causality (Wakeford 2015), and have been included in the IPCS framework and its respective updates (Boobis et al. 2006, 2008; Meek et al. 2014a; Sonich-Mullin et al. 2001). Although the framework focuses on the relevance of the mode of action, dose–response relationships and consistency among studies are also indicated as key elements. The statistical assessment is the first step for assessing the results of the toxicity tests, and has received significant attention from both, regulatory bodies (e.g. OECD guidelines on testing and assessments of chemicals) and academics (Hothorn 2014); nevertheless, the statistical analysis should be considered part of an overall assessment. This is particularly relevant in cases such as glyphosate, where the statistical analysis is inconsistent or inconclusive, with significant differences in the trend, but not in the pair-wise analysis. Lack of consistency at similar doses in the same species and strain and lack of dose–response relationships can be observed for malignant lymphomas in mice (Tables 5, 6) and adenomas in rat (Table 6). Kobayashi et al. (2010) reviewed the grounds for considering statistically significant changes as incidental, observing similar trends for unpublished and peer-reviewed scientific publications. Lack of dose–response is reported as the main justification for disregarding the results as incidental, followed by lack of physiological/toxicological significance of the effects and the comparison with historical controls. These studies support the concern surrounding conclusions that are based only on statistical significance of increased tumour incidences in a particular study, without considerations of the biological relevance of the finding.

Although the concurrent control group is always the most relevant comparator, the use of historical control data, also in combination with background incidental lesions (McInnes and Scudamore 2014), can be essential in cases of equivocal results to detect both, false positive and false negative situations. In addition to best practices (Greim et al. 2003; Keenan et al. 2009), graphical visualisations (Elmore and Peddada 2009) and statistical approaches (Dinse and Peddada 2011; Peddada et al. 2007) have been developed, although direct comparison with the historical control range in the test laboratory around the time of the study is the approach mostly used in the regulatory context, and preferred in the EU assessment. This approach was considered for malignant lymphomas and haemangiosarcomas in mice when the studies reported the historical range for the test laboratory.

Excessive toxicity, for instance toxicity at doses exceeding the MTD, can cause effects such as cell death (necrosis) with associated regenerative hyperplasia, which in turn can lead to tumour development as a secondary effect, unrelated to the intrinsic potential of the substance itself to cause tumours at lower and less toxic doses (European Chemicals Agency 2015; Knight et al. 2006). Also in the assessment of cell proliferation as mode of action for non-genotoxic carcinogens, systemic toxicity and overt cytotoxicity in the target tissue should be avoided (Wood et al. 2015). It has been suggested that almost all chemicals, including those non-genotoxic and without structural alerts for carcinogenicity, would produce statistically significant trends if tested at or above the MTD in a sufficiently large number of animals (Gaylor 2005). Significant trends for tumour induction were observed in two mouse studies but only at very high doses, well above the proposed top dose for carcinogenicity studies (OECD 2012) of 1000 mg/kg bw per day; clear indications of toxicity were observed at these high doses, such as reduced body weight, histopathological changes in the bladder and liver, and other toxic signs; consequently, the tumour induction trends were considered confounding effects due to excessive toxicity.

#### Mechanistic assessment

The relevance of the mode of action for humans constitutes the basis of the IPCS framework (Boobis et al. 2006, 2008; Meek et al. 2014a; Sonich-Mullin et al. 2001). Mode of action is defined as a biologically plausible series of key events leading to an effect (Sonich-Mullin et al. 2001) and involves interdependent networks of events with feedback loops. Differences in networks between and within human and animal populations account, in part, for interspecies differences and human variability (Meek et al. 2014a). Current approaches explore the applicability of the Adverse Outcome Pathway approach (Collier et al. 2016; Edwards et al. 2016; Zhou 2015) as a framework for linking the initial molecular interactions with the tumour promotion though plausible key events (Becker et al. 2015; Downes and Foster 2015). As the EU evaluation concluded that the incidences were due to chance and bias and the evidence does not indicate that glyphosate is an animal carcinogen, no further assessment of relevance for humans was required.

IARC, with a different focus, not targeted to individual chemicals but to a broad range of agents, has recently developed a new weight of evidence scheme, by extracting the “key characteristics” from the physical/chemical/biological/behavioural agents classified by IARC in category 1 (Smith et al. 2016). These key characteristics are defined as common properties, not to be considered mechanisms of Adverse Outcome Pathways, although are postulated as a method to synthesize information and develop adverse outcome networks. The ten characteristics are the abilities of an agent to: (1) act as an electrophile either directly or after metabolic activation; (2) be genotoxic; (3) alter DNA repair or cause genomic instability; (4) induce epigenetic alterations; (5) induce oxidative stress; (6) induce chronic inflammation; (7) be immunosuppressive; (8) modulate receptor-mediated effects; (9) cause immortalization; and (10) alter cell proliferation, cell death, or nutrient supply. It should be noted that this new approach has been applied to the recent IARC monographs, including the assessment of glyphosate.

#### Genotoxicity

The EU evaluation considers *in vitro* genotoxicity tests and *in vivo* studies performed in mammals, as those are considered to be more relevant for the assessment of the risk to humans (Yauk et al. 2015). Sixteen *in vivo* studies in somatic cells and two *in vivo* studies on germ cells were reported on rodents orally treated with dose levels up to 5000 mg/kg bw, or via intraperitoneal injections. All studies were conducted according to internationally validated guidelines; some non-GLP published studies gave negative results, while two non-GLP studies were positive in mice treated intraperitoneally with dose levels in the range of the intraperitoneal LD_50_ for mice, one study presenting major flaws. No genotoxic effects on germ cells were detected in rats or mice treated orally at dose levels up to 2000 mg/kg bw. The induction of DNA strand breaks observed in mice treated intraperitoneally with doses close to or in excess of the LD_50_ has been associated to secondary effects of cytotoxicity (JMPR 2006; Kier 2015). Modes of action associated with secondary cytotoxicity should be excluded from the assessment of the intrinsic genotoxicity potential (Bryce et al. 2014; Kitamoto et al. 2015).

IARC combines information on glyphosate and glyphosate-based formulations, compiling studies on humans, other mammals, other vertebrates, invertebrates, and plants. Regarding *in vivo* mammalian studies, IARC reports positive effects for 5 out of 11 studies; four negative studies on micronucleus formation and dominant lethal mutation reported by JMPR (2006) are not included in the IARC evaluation. Positive effects are described only for intraperitoneal administrations at doses of 300 mg/kg bw. Although these effects had been previously postulated as secondary to (cyto)toxicity (Heydens et al. 2008; JMPR 2006), the role of (cyto)toxicity is not discussed in the IARC monograph. Positive effects are mostly observed in the liver, an organ that is considered inappropriate for assessing *in vivo* genotoxic effects after intraperitoneal administration (JMPR 2006).

A recent meta-analysis on micronuclei frequency (Ghisi et al. 2016) has confirmed that positive effects are limited to intraperitoneal administrations, and that the response is much higher for glyphosate-based formulations than for the active substance. Cytotoxicity of the surfactants added to the formulations is presented as a plausible explanation, while the cytotoxicity of glyphosate in intraperitoneal administrations at high doses is not discussed. Significant differences are observed for males but not for females, a general difference is reported in the comparison of mammalian and non-mammalian systems, although similar responses are observed for mice and crocodilians (Ghisi et al. 2016).

#### Non-genotoxic modes of action

Non-genotoxic modes of action for carcinogenicity are assumed for about 9% of IARC classifications (Hernandez et al. 2009) and include endocrine disruption, tumour promotion, tissue-specific toxicity and inflammation, cytotoxicity and immune suppression, inhibition of gap-junction intercellular communications (GJICs), and other mechanisms (Benigni et al. 2013; Hernandez et al. 2009).

In the EU evaluation, the lack of evidence for carcinogenic potential of glyphosate meant that no further thought regarding the mode of action was considered necessary. IARC assessed the “key characteristics of human carcinogens” (Smith et al. 2016), concluding that there is weak evidence for receptor-mediated effects, cell proliferation or death, and immune effects, and strong evidence of oxidative stress.

### Role of surfactants and other co-formulants

The EU assessment focuses on glyphosate, aiming to establish the properties of the active substance to be considered in the assessment of each formulation by individual Member States. IARC has a different approach, addressing both glyphosate and its formulations. The potential role of the co-formulants, which differ among formulations, is not assessed; however, the IARC monograph reports a large number of mechanistic studies with negative results for glyphosate but positive results for glyphosate-based formulations, as well as differences between formulations containing similar concentrations of glyphosate, indicating that other ingredients could lead to the effects observed when testing formulations (Coalova et al. 2014; Cox and Surgan 2006). Similar results are observed for other pesticides and particularly for herbicides (Cavas 2011); this is not surprising, as the mode of action leading the herbicidal activity is usually not linked to the toxicological profile in mammals.

Surfactants are frequently used in herbicide formulations, including glyphosate. Polyethoxylated tallowamines are several orders of magnitude more cytotoxic than glyphosate (Mesnage et al. 2013); the mode of action is cell death with inhibition of the mitochondrial succinate dehydrogenase activity and membrane damage leading to necrosis. This mode of action is different from glyphosate, while similar to that observed for glyphosate-based formulations (Benachour and Seralini 2009). These tallowamines also produce oxidative and DNA damage (Nobels et al. 2011), and increase the apoptotic potential of glyphosate (Kim et al. 2013). Other surfactants as well as solvents used in pesticides formulations are cytotoxic and, possibly, genotoxic (Nobels et al. 2011).

The cytotoxicity and potential genotoxicity of other ingredients should be considered before assuming that the effects observed for a formulated product are linked to the active substance. Secondary genotoxic effects produced by cytotoxicity should also be distinguished from true genotoxic potential (Bryce et al. 2014; Kitamoto et al. 2015). In fact, the UN and EU guidance recommends carcinogenicity and genotoxicity studies to be conducted on individual chemicals, limiting testing of mixtures/formulations to cases where synergistic effects are expected (United Nations 2015).

### From hazard assessment to public health risk assessment

While IARC focuses exclusively on the hazard identification, regulatory assessments also include the estimation of the toxicological potency of the substance and the setting of toxicological reference values to be used in the human health risk assessment. The toxicological reference values offer quantitative indications of the toxicity of a chemical, indicating the levels of human exposure that, according to the current scientific knowledge, are considered acceptable from a regulatory perspective. The recent EFSA evaluation has changed significantly the toxicological profile of glyphosate, compared to the previous EU assessment (Table 8).

**Table 8:** Summary of the recent EU toxicological assessment of glyphosate and derivation of reference doses of risk assessment

	Relevant endpoints mg/kg body weight (per day)	Uncertainty factor	Reference dose for risk assessment mg/kg bw (per day)
Chronic dietary toxicity	Rat overall NOAEL: 100 Mice overall NOAEL: 150 Rodent reproductive NOAEL: 300 Rat neurotoxicity NOAEL: 617 Dog short-term NOAEL: 300 Critical endpoint: Rabbit NOAEL: 50 (maternal and developmental, also relevant for short-term exposures)	100	Acceptable Daily Intake (ADI): 0.5
Acute dietary toxicity		100	Acute Reference Dose (ARfD): 0.5
Chronic non-dietary toxicity		100 × 5 (accounting for 20% oral absorption)	Acceptable Operator Exposure Level (AOEL): 0.1

**Fig. 1:**
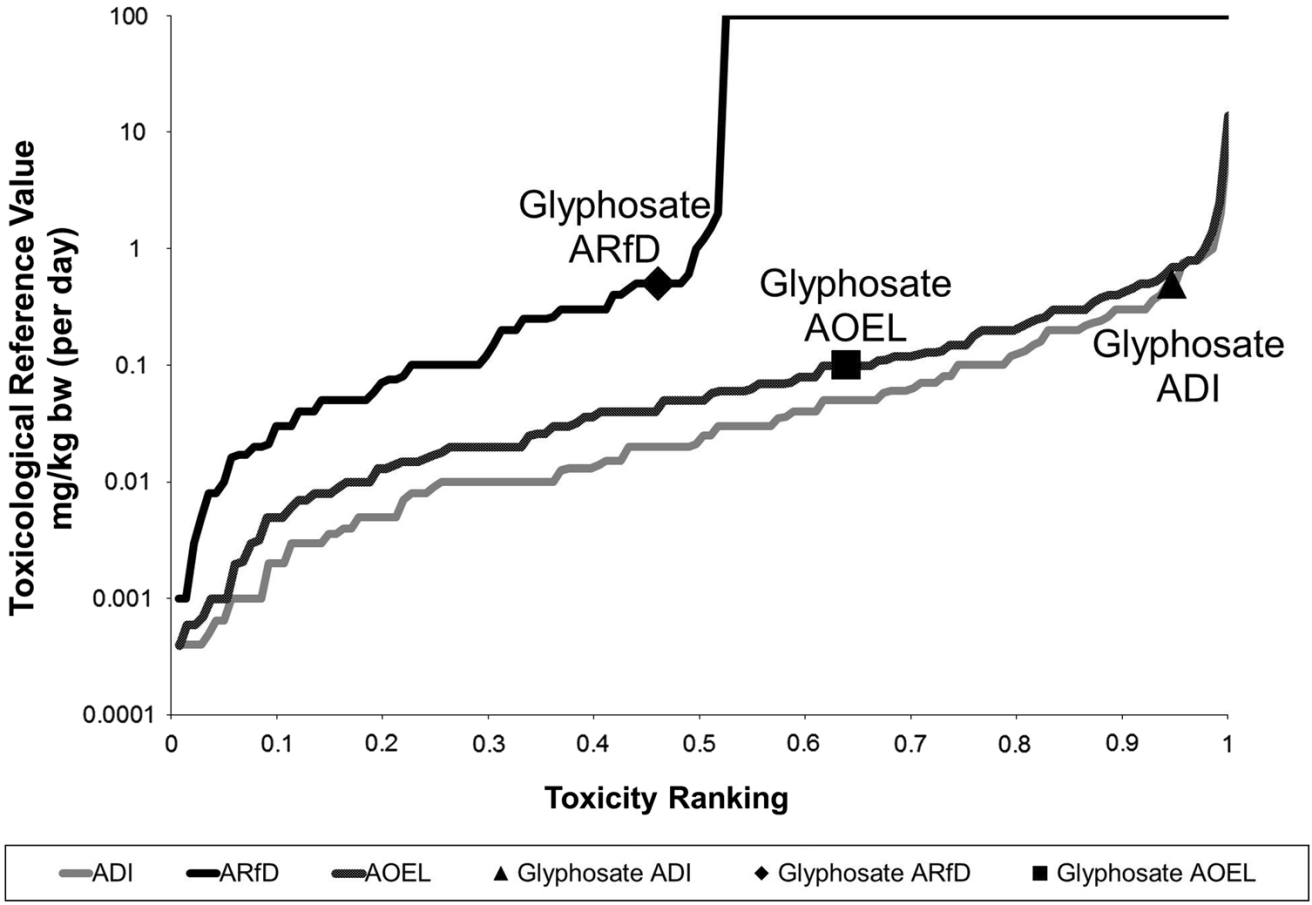
Graphical representation of the EFSA proposed changes in the glyphosate toxicological profile expressed as the relative toxicity ranking. This ranking represents the percentile of each glyphosate’s Toxicological Reference Value within the distribution of 141 herbicides assessed in the EU (data extracted from the EU pesticides database. http://ec.europa.eu/food/plant/pesticides/eu-pesticides-database/public/?event=activesubstance.selection&language=EN on 25 May 2016)

The Acute Reference Dose (ARfD) and Acceptable Daily Intake (ADI) represent oral doses that should not be exceeded in a single event (or repeated within 24 h) or daily in long term exposures, respectively. The Acceptable Operator Exposure Level (AOEL) represents a systemic daily dose that should not be exceeded in non-dietary exposures. Figure 1 visualises the current and previous EU toxicological reference values for glyphosate, compared with those established for the entire group of herbicides assessed in the EU. The ranking and percentile within the distribution of ca. 150 herbicides assessed in the EU (data extracted from the EU pesticides database http://ec.europa.eu/food/plant/pesticides/eu-pesticides-database/public/?event=homepage&language=EN) gives an indication of the relative toxicity of glyphosate to humans compared to the other herbicides. In contrast with previous evaluations, effects produced after acute exposures were considered relevant, requiring an ARfD and an acute risk assessment (European Food Safety Authority 2015b). The human, animal and mechanistic evidence indicates that glyphosate cannot be considered as a potent DNA reactive tumour-initiating chemical, and that a risk assessment based on threshold toxicological reference values is scientifically valid (SCOEL 2013). The data summarised in Tables 4, 5 and 6 confirms that the proposed reference values (Table 8) provide sufficient protection for all effects observed in the carcinogenicity and long-term toxicity studies, including the trends for tumour induction considered as sufficient evidence by IARC.

Glyphosate has a relative low long-term dietary toxicity, being within the 10% of herbicides with higher ADI. Regarding short-term dietary exposure, the EU assessment proposed an ARfD which ranks glyphosate as slightly more toxic (45th percentile) than the average for herbicides. This new toxicological profile requires the re-assessment of health risks, which had only considered chronic exposure until now (Shao-Wen and Chun-Hong 2015). The need for personal protective equipment for glyphosate applicators is identified in the EFSA Conclusion. The need for an ARfD triggers also new considerations regarding the role of sporadic AOEL exceedance when addressing the risk of short-term inhalation and dermal exposures during application, including bystander and resident exposure in aerial applications, which are standard practice outside the EU in forest (Rolando et al. 2013) and for the control of illegal crops (Benner et al. 2016). Exposure estimations for children entering the area after application (Solomon et al. 2007) are higher than the proposed toxicological threshold.

**Fig. 2:**
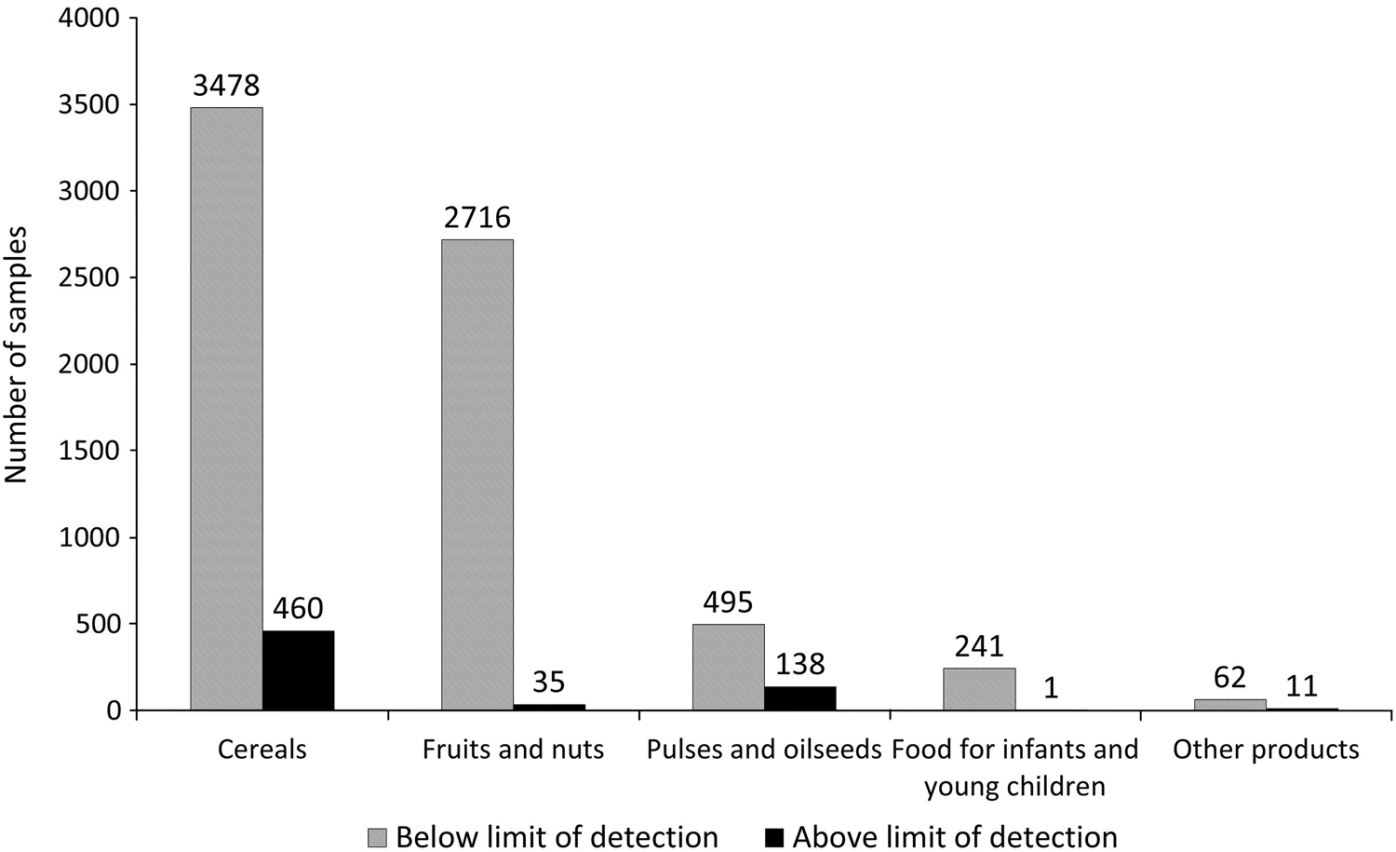
Summary of EU monitoring data on glyphosate residues in food (2012–2014)

Regarding residues in food, a comprehensive update of the dietary risk assessment will be performed in the EU, following the decision on the approval of glyphosate, covering all EU uses and the residues expected on imported food. Meanwhile, Niemann et al. (2015) have compiled information on human biomonitoring data, and concluded that current exposures are well below the toxicological references values; exposure of European citizens seems to be lower than that of Americans. To complement these estimations, an indicative consumer exposure assessment based on EU monitoring data for glyphosate residues in food generated by competent authorities in the EU Member States is described below. The assessment covers over 10,000 samples of different types of food analysed for glyphosate residues between 2012 and 2014 (Fig. 2). Member States focussed the control activities for glyphosate mainly on crops relevant for human consumption, where the presence of glyphosate was expected, such as cereals (almost 4000 samples), followed by fruits, vegetables, pulses and oilseeds; it should be noted that only limited information is available on feed products such as soya beans (only nine soya beans samples were analysed). Overall glyphosate was detected in 6.3% of the samples, mostly in cereals (11.7% of the samples analysed contained residues above the Limit of Quantification), but also in lentils, linseed and table grapes, mostly from outside the EU. The legal limits were exceeded in 0.2% of the samples analysed for glyphosate. A very conservative risk assessment screening has been conducted with the EFSA PRIMO model (European Food Safety Authority 2007), using conservative assumptions. Table 9 summarises the residue levels measured in food items which were identified as main contributors in the risk assessment using European food consumption data. The data have been extracted from the EU pesticides residues monitoring programme (European Food Safety Authority 2016). Detailed information is provided in the supporting information.

**Table 9:** Glyphosate residue levels reported for the food items contributing with over 0.1% of the ADI or 2% of the ARfD in the European consumers’ risk assessment (EFSA 2016)

Food item	Number of samples analysed for glyphosate	Percentage of samples with residues > LOQ	Maximum level mg/kg	Mean value mg/kg
Apples	215	1.9	0.10	0.02^$^
Barley	188	18.6	8.00	0.24
Beans (dry)	132	11.4%	4.00	0.16
Beans (with pods)	123	0.8%	0.05	0.02^$^
Lentils (dry)	277	30.3%	19.00	0.59
Oranges	192	0.5%	0.10	0.03^$^
Peas (dry)	41	37.7%	6.39	0.59
Peas (with pods)	38	7.9%	1.40	0.13
Peas (without pods)	22	0%	0.10	0.04^$^
Potatoes	88	0%	0.10	0.02^$^
Rye	557	4.1%	3.40	0.13
Wheat	2318	13.2%	4.00	0.14

^$^The mean value is similar to the Limit of Quantification

The acute risk assessment used the maximum reported result. The chronic risk assessment used mean residue concentrations, assuming that residues below the Limit of Quantification (LOQ) actually occurred in concentrations equivalent to the LOQ; considering that over 94% of the samples analysed did not contain residues above the LOQ, this assumption contributes to the conservatism of the estimated exposure. The chronic exposure was well below the ADI (0.5% for unprocessed products and 0.6% of the ADI when processed foods are included). In the acute risk assessment, the highest exposure was calculated for lentils (23.4% of the ARfD), followed by beans (14.6%) and wheat (11.6%). Pending on the on-going EFSA assessment, these estimations further support the conclusion that glyphosate residues in food do not represent a public health concern for European citizens.

## Conclusions

The following main factors should be considered when explaining the differences between IARC and the EU evaluations: the evidence and information sources, the methodology and the overall aim. The comparison is summarised in Table 10.

**Table 10:** Comparative summary of IARC and EU assessments and conclusions

Issue	IARC	EU regulatory assessment
Epidemiological studies		
Evidence	Same human evidence based on published epidemiological studies. Different animal and mechanistic conclusions in the plausibility assessment	
Assessment	Positive and negative associations. Associations considered biologically plausible	Positive and negative associations. Associations considered week and lacking biological plausibility
Conclusion	Sufficient for “Limited evidence” in humans	Contradictory evidence, insufficient to be considered as “limited evidence”
Animal carcinogenicity		
Evidence (see Table 4)	US EPA and JMPR reports summarising industry studies results	Full industry study reports, covering a larger data set for mice and rats
Assessment (see Tables 5, 6)	Positive trends in one sex in some studies. Pair-wise comparisons without dose–response. No indication on consistency assessment between studies, sexes or consideration of excessive toxicity	Large data set with mostly negative findings. Positive findings were inconsistent (between sexes, statistical approaches, and among studies), observed only at very high doses above the Maximum Tolerable Dose, or lack of dose response
Conclusion	Sufficient evidence for carcinogenicity in animals	Unlikely to be carcinogenic in animals according to UN GHS weight of evidence
Genotoxicity		
Evidence	5 published *in vivo* studies on mammals, 1 secondary reference to industry studies and studies on formulations. Large coverage of non-mammalian species and formulations	Focus on 16 *in vivo* studies on mammals; guideline studies supported by additional published studies. Assessment limited to mammals and glyphosate active substance
Assessment	Biomarkers of DNA adducts and various types of chromosomal damage generally positive in the liver but only at high intraperitoneal doses (300 mg/kg bw) with mixed results for the kidney and bone marrow Inconsistent effects between glyphosate and glyphosate formulations reported for several studies, but not further assessed	Positive clastogenic effects in 2 out of 6 intraperitoneal studies at high toxic doses (above i.p. LD_50_) in studies showing methodological deficiencies. 1 weak positive out of 8 oral studies limited to high dose, one sex, and high SD Positive results in indicative studies such as DNA strand breaks do not detect mutagenicity, rather cytotoxicity Consistent negative results for gene mutation in both bacteria and mammalian cells
Conclusion	Strong evidence that exposure to glyphosate is genotoxic	Unlikely to be genotoxic in humans. No classification for mutagenicity
Overall conclusion on carcinogenicity		
Hazard	Probably carcinogenic in humans. IARC Group 2A	Unlikely to be carcinogenic in humans. No classification as carcinogen

*SD* standard deviation

### Evidence in humans

The same epidemiological studies were used in both assessments; all studies focussed on farmers exposed to formulations. For pesticides, the regulatory dossier may include information on medical surveillance and epidemiological studies on manufacturing plant personnel directly exposed to the active substance; but this was not the case for glyphosate. The key IARC role in compiling and evaluating human evidence is well proven, and the EU assessment was updated to consider recent publications included in the IARC monograph. The same weak evidence in humans for the carcinogenicity of glyphosate was interpreted differently by IARC and EFSA. IARC considered the association between exposure to glyphosate and non-Hodgkin lymphoma as “limited evidence in humans”; while in the EU assessment, most experts considered the evidence as “very limited” and insufficient for triggering the classification. The difference in the interpretation between IARC and the EU is mainly related to the fact that IARC is because IARC considered that glyphosate is carcinogenic in animals, and concluded that strong evidence for two mechanisms, genotoxicity and oxidative stress, supported the plausibility of the weak association in humans.

### Evidence on carcinogenicity in experimental animal models

Regarding animal carcinogenicity, three main aspects should be considered for understanding the different conclusions from IARC and EFSA. Lack of consistency among studies on the same species and strain at equivalent doses supported the conclusion of chance results in the EU evaluation. IARC, however, could not use some studies included in the EU evaluation, since the EU assessment was on-going and only a draft was available at the time of the IARC Working Group meeting, limiting the capacity for checking consistency among studies. Second, the lack of consistency between sexes; according to the UN-GHS criteria, a plausible sex-related mechanism should be investigated in these cases, and was not identified in the EU assessment. No specific guidance is provided in the IARC evaluation and no indication is provided in the monograph. Third, the role of secondary effects observed at doses with excessive toxicity. For regulatory assessments, when classification is linked to labelling and risk management options, secondary effects due to excessive doses are excluded as the assessment focuses on the intrinsic capacity of the chemical to induce tumours at lower, less toxic doses. This element is not described in the IARC methodology, and the IARC Working Group considered as positive trends those triggered by tumour incidences at doses with demonstrated excessive toxicity. Regulatory assessments have access to full study reports; for IARC, unpublished industry-sponsored studies are secondary information sources, and their use is limited to the study summaries from previous assessments published by other agencies. Despite not having access to the original study reports, the IARC Working Group was able to run new statistical analyses, although its capacity for verifying details relevant for assessing the biological relevance was limited by the level of detail provided in the reports published by the regulatory agencies. The comparison with the WHO expert group JMPR assessments for glyphosate, conducted in 2004 and 2016, is informative regarding the value of granting the experts access to the full study reports.

### Evidence on genotoxicity and other mechanisms of carcinogenicity

Regarding sources of mechanistic information, genotoxicity/mutagenicity should be discussed independently of other possible mechanisms. As observed for glyphosate, both industry-sponsored and scientific publications offer relevant information on the genotoxicity potential of pesticides that has raised interest among the scientific community. On one hand, IARC included one industry-sponsored study reported by the US-EPA but not those reported by JMPR (JMPR 2006); on the other hand, IARC reviewed effects observed in non-mammalian systems, which were considered of limited relevance for the assessment of carcinogenicity in humans in the regulatory assessment. IARC also assessed glyphosate-based formulations.

An important difference among IARC and regulatory assessment is the identification of a non-threshold genotoxic mode of action for carcinogenicity. This is not part of the IARC evaluation, while for regulatory assessment this is a key element triggering the risk assessment methodology. The IARC monograph used genotoxicity and oxidative stress as supporting mechanistic evidence; according to IARC principles, no indication is provided regarding threshold or non-threshold modes of action. The IARC allocation in group 2A may suggest that for the IARC Working Group the evidence on genotoxicity was insufficient for considering glyphosate as a potent DNA reactive non-threshold genotoxic human carcinogen. In fact, all oral studies, even at very high doses, are negative and the only *in vivo* mammalian positive evidence was for intraperitoneal studies at very high doses at which (cyto)toxicity is expected. This is again linked to the consideration of secondary effects due to severe systemic toxicity described above for the animal studies, which should be excluded for the classification of genotoxicity and carcinogenicity according to the UN-GHS criteria.

Other mechanistic studies should be discussed in connection with the methodological approach. With the exception of genotoxicity, mechanistic data on the mode of action are used in the regulatory context for assessing the relevance for humans, and are mostly used to downgrade the classification (Boobis et al. 2006; Clewell 2005; Meek et al. 2014a). Mechanistic data can be pivotal in IARC evaluations with inconclusive evidence in humans (Cogliano et al. 2008; Lauby-Secretan et al. 2016); and IARC has used mechanistic data for upgrading 52 agents and downgrading 8 agents (Cogliano et al. 2008). The recent review of the IARC approach for assessing mechanistic information may further change this picture. Strong evidence on non-genotoxic mechanisms is included in the recent IARC assessments for lindane, DDT and 2,4-D (Loomis et al. 2015). Moreover, mechanistic information is essential in the assessment of causality *versus* chance and bias.

To summarise, definitions for limited and sufficient evidence in humans and animals are identical for IARC and the UN-GHS; however, differences in criteria and methodological considerations for weighing and assessing the evidence can lead to divergent interpretations between the IARC assessment and regulatory evaluations following the UN-GHS criteria, even when based on the same evidence.

The differences between IARC and regulatory assessments are related not only to parallel historical developments, but to the different overall scope. IARC classifications represent a first step, alerting on the carcinogenicity potential of a broad range of agents; scientific regulatory assessments are connected to specific risk management recommendations, such as labelling, packaging requirements, use restrictions, etc., and produce the basis to be used in the risk assessment. In this different context, the focus and role of conservativeness is very different. While IARC assessments are not connected to risk management decisions, and are based exclusively on published information, without access to the full study reports for regulated products, regulatory assessments may identify data gaps and request additional studies to confirm or exclude potential concerns identified during their evaluation.

Human health safety is a critical issue for understanding the consequences of scientific divergences regarding the carcinogenicity classification of glyphosate. Regulatory assessments cover all relevant effects, not only carcinogenicity. Effects other than tumour induction were responsible for setting the NOAELs of the long-term toxicity–carcinogenicity studies, and the toxicological reference values were established from critical effects observed at lower dose levels in other studies. From a health assessment perspective, the IARC-EFSA scientific divergence is at lower dose levels that are in reality of limited, if any, relevance. The toxicological reference values proposed by EFSA provide a margin of protection of about four orders of magnitude for the trends in tumour induction and genotoxic damage at toxic levels reported by IARC. Those effects are expected only in concomitance with other signs of toxicity and at exposure levels orders of magnitude higher than the toxicological reference values recommended by EFSA. Risk assessments based on human biomonitoring and monitoring of levels of glyphosate residues in food have not identified concerns for consumers, and a full consumers’ risk assessment of all EU uses is on-going.

## Electronic supplementary material

Below is the link to the electronic supplementary material.

Supplementary material 1 (DOCX 58 KB)
