# Supplementary material for: Glyphosate toxicity and carcinogenicity: a review of the scientific basis of the European Union assessment and its differences with IARC
Source: Arch Toxicol. 2017 Apr 3;91(8):2723–43. doi: 10.1007/s00204-017-1962-5 (PMC5515989; doi:10.1007/s00204-017-1962-5)
Supplement: Supplementary file 1 — Supplementary material 1 (DOCX 58 KB) [file 204_2017_1962_MOESM1_ESM.docx]

**Supplementary material**

Table S1. Detailed comparison of EU and IARC classifications for the pesticide active substances assessed by EFSA. The EU information was extracted from the European Commission Pesticides Database: <http://ec.europa.eu/food/plant/pesticides/eu-pesticides-database/public/?event=activesubstance.selection&language=EN>, in January 2016; the IARC information was extracted from the IARC web site and kindly reviewed by IARC (the authors acknowledge the revision conducted by IARC staff).

| **Substance** |  | | **IARC available, year** |
| --- | --- | --- | --- |
|  | **EU** | |  |
|  | **(Reg. 1272/2008)** | |  |
| **Pesticide active substances with “stricter” IARC classification** | | | |
| 2,4, D | none | | Group 2B, in prep |
| DDT | 2 | | Group 2A, in prep |
| Diazinon | none | | Group 2A, 2015 |
| Ethylene oxide | 1B | | Group 1, 2012 (upgraded) |
| Formaldehyde | 1B | | Group 1, 2012 |
| Glyphosate | none | | Group 2A, 2015 |
| Lindane | none | | Group 1, in prep |
| Malathion | none | | Group 2A, 2015 |
| ^[[1]](#footnote-1)^MCPA[1] | none | | Group 2B, 1987 |
| Parathion | none | | Group 2B, 2015 |
| Tetrachlorvinphos | none | | Group 2B, in prep |
| **Pesticide active substances with “stricter” EU classification** | | | |
| Aldrin | 2 | | Group 3, 1987 |
| Anthracene oil | 1B | | Group 3, 2010 |
| Captan | 2 | | Group 3, 1987 |
| Carbaryl | 2 | | Group 3, 1987 |
| Chlorpropham | 2 | | Group 3, 1987 |
| Di-allate | 2 | | Group 3, 1987 |
| Dieldrin | 2 | | Group 3, 1987 |
| Hexachlorobenzene | 1B | | Group 2B, 2001 |
| Monuron | 2 | | Group 3, 1991 |
| ^[[2]](#footnote-2)^Nitrofen[2] | 1B | | Group 2B, 1987 |
| ^[[3]](#footnote-3)^p-Chloronitrobenzene[3] | 2 | | Group 3, 1996 |
| Simazine | 2 | | Group 3, 1999 |
| Thiourea | 2 | | Group 3, 2001 |
| Trifluralin | 2 | | Group 3, 1991 |
| **Pesticide active substances with “equivalent” classifications for IARC and EU** | | | |
| Aldicarb | none | | Group 3, 1991 |
| Amitrole | none | | Group 3, 2001 |
| Atrazine | none | | Group 3, 1999 |
| Captafol | 1B | | Group 2A, 1991 |
| Chlordane | 2 | | Group 2B, 2001 |
| Chlordecone | 2 | | Group 2B, 1987 |
| Chlorobenzilate | none | | Group 3, 1987 |
| Chlorothalonil | 2 | | Group 2B, 1999 |
| Deltamethrin | none | | Group 3, 1991 |
| Dicofol | none | | Group 3, 1987 |
| Endrin | none | | Group 3, 1987 |
| Fenvalerate | none | | Group 3, 1991 |
| Ferbam | none | | Group 3, 1987 |
| Fluometuron | none | | Group 3, 1987 |
| Heptachlor | 2 | | Group 2B, 2001 |
| Maneb | none | | Group 3, 1987 |
| Methoxychlor | none | | Group 3, 1987 |
| Methylbromide | none | | Group 3, 1999 |
| Naphthalene | 2 | | 2B, 2002 |
| Ortho-phenyl phenol | none | | Group 3, 1999 |
| p-Dichlorobenzene | 2 | | Group 2B, 1999 |
| Pentachlorophenol | 2 | | Group 2B, 1999 |
| Permethrin | none | | Group 3, 1991 |
| Picloram | none | | Group 3, 1991 |
| Quintozene | none | | Group 3, 1987 |
| Thiram | none | | Group 3, 1991 |
| Trichlorfon | none | | Group 3, 1987 |
| Zineb | none | | Group 3, 1987 |
| Ziram | none | | Group 3, 1991 |
| **Pesticide active substances for which only one assessment is available** | | | |
| 1,2-Dibromoethane | 1B | | No |
| Aclonifen | 2 | | No |
| Alachlor | 2 | | No |
| Aramite | no data available | | Group 2b, 1987 |
| Bifenthrin | 2 | | No |
| Camphechlor | 2 | | No |
| Carbon monoxide | 1B | | No |
| Chlorotoluron | 2 | | No |
| Chlozolinate | 2 | | No |
| Cinidon ethyl | 2 | | No |
| Dichlorvos | no data available | | Group 2B, 1991 |
| Dimoxystrobin | 2 | | No |
| Diuron | 2 | | No |
| Epoxiconazole | 2 | | No |
| Etridiazole | 2 | | No |
| Fenoxycarb | 2 | | No |
| Fentin acetate | 2 | | No |
| Fentin hydroxide | 2 | | No |
| Flusilazole | 2 | | No |
| Folpet | 2 | | No |
| Forchlorfenuron | 2 | | No |
| Fuberidazole | 2 | | No |
| Furmecyclox | 2 | | No |
| Iprodione | 2 | | No |
| Isoproturon | 2 | | No |
| Kresoxim-methyl | 2 | | No |
| Linuron | 2 | | No |
| Mepanipyrim | 2 | | No |
| Metazachlor | 2 | | No |
| Mirex | not assessed | | Group 2B, 1987 |
| Molinate | 2 | | No |
|  |  | |  |
| Paraffin oil/(CAS 64741-88-4) | 1B | | No |
| Paraffin oil/(CAS 64741-89-5) | 1B | | No |
| Paraffin oil/(CAS 64741-97-5) | 1B | | No |
| Paraffin oil/(CAS 64742-54-7) | 1B | | No |
| Paraffin oil/(CAS 64742-55-8) | 1B | | No |
| Paraffin oil/(CAS 64742-65-0) | 1B | | No |
| ^[[4]](#footnote-4)^Petroleum oils/(CAS 74869-22-0))[4] | 1B | | No |
| ^[[5]](#footnote-5)^Phenols[5] | 1B | | No |
| Profoxydim | 2 | | No |
| Propargite | 2 | | No |
| Propazine | 2 | | No |
| Propham | no data available | | Group 3, 1987 |
| Propyzamide | 2 | | No |
| Proquinazid | 2 | | No |
| Pymetrozine | 2 | | No |
| Sodium ortho-phenylphenate | not assessed | | Group 2B, 1999 |
| Sulfallate | not assessed | | Group 2B, 1987 |
| Tar acids | 1B | | No |
| Tepraloxydim | 2 | | No |
| Tralkoxydim | 2 | | No |
| Vinclozolin | 2 | | No |
|  |  | |  |
|  |  | |  |
| ^[[6]](#footnote-6)^[1] Evaluated by IARC only as part of the class cholorophenoxy herbicides, also including 2,4-D, 2,4,5-T, dichlorprop, and mecoprop. | | | |
| ^[[7]](#footnote-7)^[2] IARC evaluated the technical-grade. | |  | |
| ^[[8]](#footnote-8)^[3] Considered by IARC as a chemical used in pesticide production, not as a pesticide itself. | | | |
| ^[[9]](#footnote-9)^[4] IARC evaluated Petroleum refining (occupational exposures in) Group 2A, 1989. | | | |
| ^[[10]](#footnote-10)^[5] Phenols were evaluated by IARC as: Pentachlorophenol/Polychlorophenols and their sodium salts and Chlorophenols: Group 2B, 1999. | | | |
|  | | | |
|  | |  | |
|  | | | |

Table S2. Study reference used for this publication, study authors and year, reference used for citing the study in the EU assessment, reference used in the IARC assessment, and pages in the Glyphosate Summary Dossier (SD) available in the EFSA website and in the EFSA background document (Germany, 2015) were the description of the study by the notifier and by the Rapportuer Member State (RMS) are available.

| **Study Reference - Authors (year)** | **Study reference in EU assessment** | **Study reference in IARC assessment** | **Study detailed descriptions** *by notifier (pages in Summary Dossier (SD)) and RMS (pages in Germany 2015 (RAR))* |
| --- | --- | --- | --- |
| **Mice long-term chronic toxicity and carcinogenicity studies used in the EU evaluation** | | | |
| A - Knezevich and Hogan (1983) | TOX9552381, 1983 | US-EPA 1985a,b, 1986, 1991a | *Described in the previous EU evaluation US-EPA and IARC* |
| B - Atkinson et al. (1993) | TOX9552382, 1993 | JMPR 2006 | *Described in the previous EU evaluation JMPR and IARC* |
| C - Sugimoto (1997) | IIA, 5.5.3/03  ASB2012-11493, 1997, | not assessed by IARC | *SD pp 516-525*  *RAR pp 1030-1040* |
| D - Wood et al. (2009) | IIA, 5.5.3/02  ASB2012-11492, 2009 | not assessed by IARC | *SD pp 511-516*  *RAR pp 1023-1030* |
| **Rat long-term chronic toxicity and carcinogenicity studies used in the EU evaluation** | | | |
| E - Lankas (1981) | IIA, 5.5.2/05  TOX2000-595, 1981 | IARC: US-EPA 1991a,b.c.d | *SD pp 479-485*  *RAR pp 987-993* |
| F - Stout and Ruecker (1990) | IIA, 5.5.2/06  TOX9300244, 1990 | US-EPA 1991a,b.c.d | *SD pp 485-491*  *RAR pp 993-999* |
| G - Atkinson et al. (1993) | IIA, 5.5.2/04  TOX9750499, 1993 | JMPR,2006 | *SD pp 471-478*  *RAR pp 999-1007* |
| H - Suresh (1996) | IIA, 5.5.2/01  TOX9651587 | not assessed by IARC | *SD pp 451-456*  *RAR 1007-1013* |
| I - Lankas 1997 | IIA, 5.5.1/01  TOX2000-1998, 1996 | JMPR,2006 | *SD pp 447- 451;*  *RAR pp 955-960* |
| J - Enomoto (1997) | IIA, 5.5.2/02  ASB2012-11484, 1997, | not assessed by IARC | *SD pp 457-463*  *RAR pp 960-966* |
| K - Brammer (2001) | IIA, 5.5.2/03  ASB2012-11488, 2001, | JMPR,2006 | *SD pp 463-471*  *RAR pp 972-980* |
| L - Wood et al. (2009) | IIA, 5.5.2/08  ASB2012-11490 | not assessed by IARC | *SD pp 496-502*  *RAR pp 980-987* |
| M - Chruzielska *et al*., 2000, | IIA, 5.5.3  ASB2013-9829 Chruzielska et al., 2000 | Chruzielska et al., 2000 | *RAR pp 533, 550-551* |
| **Industry sponsored GLP studies considered non-acceptable during the EU assessment** | | | |
| N - Kumar (2001) | IIA, 5.5.3/01  ASB2012-114912001 | not assessed by IARC | *SD pp 504-51*  *RAR pp 1013-1023* |
| O - Bhide (1997) | IIA, 5.5.2/07  ASB2012-11489 1997 | not assessed by IARC | *SD pp 491-496*  *RAR pp 967-972* |
| **Published studies conducted with glyphosate-based formulations and considered non-reliable for the assessment of glyphosate carcinogenicity during the EU assessment** | | | |
| P - George et al., 2010 | IIA, 5.5.3  ASB2012-11829  George et al., 2010 | George et al., 2010 | *RAR pp 533, 547-548* |
| Q - Seralini et al., 2012, re-published 2014 | IIA, 5.5.3  ASB2012-15514  Seralini et al., 2012 | Seralini et al., 2012 | *RAR pp 532, 548-549* |

Table S3. Detailed output of the risk for European consumers according to the EFSA PRIMo calculations. Detailed input values for residue levels in food commodities used for the EFSA PRIMo version 2 calculations are available under request. EFSA PRIMo tool is available at http://www.efsa.europa.eu/en/applications/pesticides/tools.

1. [↑](#footnote-ref-1)
2. [↑](#footnote-ref-2)
3. [↑](#footnote-ref-3)
4. [↑](#footnote-ref-4)
5. [↑](#footnote-ref-5)
6. [↑](#footnote-ref-6)
7. [↑](#footnote-ref-7)
8. [↑](#footnote-ref-8)
9. [↑](#footnote-ref-9)
10. [↑](#footnote-ref-10)
